# Thinking Slow About IP in Times of Pandemic

**DOI:** 10.1007/s40319-020-00942-x

**Published:** 2020-05-25

**Authors:** Begoña González Otero

**Affiliations:** grid.469479.1 0000 0001 1955 4116 Dr.; Senior Research Fellow, Max Planck Institute for Innovation and Competition, Munich, Germany

By the time you read these words, each of us might be in different countries, on different continents and even in different hemispheres. Despite the physical distance, our current experience could not be more alike. We are social distancing at home, we all see practically empty streets outside, we worry about the health of our friends and families, and we are trying to make sense of what is happening around us.

As *Daniel Kahneman* observed in his book *Thinking, Fast and Slow*, we have two principal modes of thinking. In a crisis, our survival instinct is to think fast, to simplify and to jump to conclusions. However, in doing so, we risk neglecting how our “think-fast world” may have ignited the flame of the COVID-19 pandemic and future crises to come, whether through questionable trade and environmental practices, or the absence of planning and underinvestment for pandemics. There has been sufficient warning about the potential for a highly infectious viral outbreak. Hence, while it is natural to think fast in survival mode, we also need to think slow, to reflect and to anticipate.

Similar fast reactions are taking place in the field of intellectual property rights. In the last two months we have seen the promotion of data collection and processing via digital public health technologies by governments and private undertakings as strategic remedies for relaxing confinement during the pandemic. Yet, as *Natali Helberger* stated: “Apps are suggested and understood by many people as the magical silver bullet to opening up society again, which they’re not. We don’t know how effective they are, we don’t know what the side effects are, and we know apps alone can’t be a solution to this.”^1^ There have also been rapid moves urging international institutions to ensure the use of intellectual property rights as a support rather than a hindrance to tackle the COVID-19 pandemic,^2^ as well as proposals directed towards undertakings to pledge to license certain intellectual property rights free of charge in the spirit of minimizing the impact of the pandemic, including a model license.^3^

As suggested by *Nassim Taleb*, narrative fallacies inevitably emerge as a result of our ongoing attempt to make sense of the world.^4^ He also insinuates that as humans, we have the tendency to condense complex realities into oversimplified stories that neatly account for results. At the present time, there does not appear to be any empirical evidence that intellectual property rights are acting as a barrier to access vital medical preventive measures such as vaccines, treatments, cures or medical devices. On the one hand, as pointed out by the South Centre, there are relevant precedents that governments and policymakers should take into account in addressing the COVID-19 pandemic, both as a tool for procurement and import of patented medicines and making use of the compulsory licensing mechanism.^5^ On the other hand, we have never witnessed such a massive open collaboration movement aiding in the fight against COVID-19. The problem is rather that there is still no vaccine or scientifically proven and approved treatment or cure available.

The main policy challenge at this time seems to be the encouragement of innovation that may lead to a vaccine and treatments and cures. Valuable studies on how to foster research and development for infectious diseases have been carried out already.^6^ Nonetheless, we also need to foster innovation that assists in managing the crisis – not only from a medical perspective – regarding labor organization, mobility, education, manufacturing, etc. More than ever, we need innovation for the common good. However, focusing only on access rather than on the encouragement of innovation may create a disincentive for investment.

In 2009, right after the financial crisis, *Angel Gurría*, OECD Secretary-General made the following statement:

One pervasive feature about the current environment is uncertainty: uncertainty about the fallout of the financial crisis on the real economy, about the appropriate response to the financial crisis, even about the economic governance of the world of tomorrow. To a large extent, these uncertainties stem from the fragilities that the financial crisis has revealed throughout the world. Contrary to past financial and economic crises, this one is truly global. Its origin was in the United States; however, this was really only the canary in the coalmine. We failed to listen to its song. It was a song of regulatory market and policy failures. That song, we now know, swiftly resonated into other financial markets and to other countries, creating the worst financial crisis since the Great Depression.^7^

Make a few small twists and replace some of the words, and it would perfectly describe our present times.

Innovation depends on trust, and trust is fragile. It also requires a favorable culture, institutional consistency and a legal system that is predictable and transparent. During a global crisis, when rapid investment is needed most, lawmakers should reaffirm their support for research-based innovation. Traditionally, situations of insufficient investment in innovation have been addressed via either direct legal market intervention, by granting aid for research and development, or by transforming the public into a private good through the grant of intellectual property rights. Over the last decades there has been increasing questioning of whether incentives for innovation can be inferred from the system of intellectual property rights. We have also seen that excessive protection can lead to dysfunctional effects. The protection of intellectual property forms part of the dynamic competition system and has to be at the service of its objectives and operation.^8^ In this system, competition law intervenes to ensure that private parties, jointly or alone, by the exercise of market power, do not go too far. As *Hanns Ullrich* noted, this intervention “is to safeguard the incentive and reward rationales of intellectual property protection while at the same time controlling the risks of an undue extension of legal exclusivity”.^9^

Nevertheless, now there is much more. This health crisis has taken place in the middle of the Fourth Industrial Revolution. And as a pandemic, it is fundamentally different from any previous revolution or crisis faced by humanity. As *Klaus Schwab* observed, the Fourth Industrial Revolution is characterized by a range of new technologies that are fusing the physical, digital and biological worlds, impacting all disciplines, economies and industries, and even challenging ideas about what it means to be human.^10^

In human history, changes have always followed one another in a linear path. In the last 30 years, these changes, as with the COVID-19 pandemic, have occurred exponentially,^11^ and as with the pandemic, we have not been ready: neither is the law ready, nor is intellectual property. One of the first changes we are experiencing is the augmented use and application of AI-based technologies in combination with connectivity (the Internet of Everything). We are observing that innovation cycles are becoming shorter, with an associated decrease in costs of experimenting in new product versions; novel opportunities for innovation in services arise, while, as the COVID-19 pandemic has exposed, innovation processes become more and more collaborative and diverse. Even from a competition perspective, the traditional market boundaries are losing relevance, or the boundaries between manufacturing and services are becoming diluted. Nevertheless, as with the current pandemic, it is the first of more to come: the use and combination of AI technologies with quantum computing, with nanotechnology, etc., are only a small sample of a snowballing list of forthcoming revolutions. We have countless challenges ahead of us. Perhaps the time has come to abandon traditional inflexible positions such as “one-size-fits-all”. Perhaps it is time to abandon the adaptation of the law as a type of response, because it may no longer be about adaptation, but about disruption and invention. What does this mean? It may be that, as with the challenges of the COVID-19 pandemic, some of the legal tools we have are no longer suitable to encourage innovation or to serve the common good in this new reality.

*Phillip Allott* once pointed out: “In law-making society speaks to its future, intending that, when the time comes, its future will listen to its past.”^12^ We now need to imagine our best future, to anticipate, to think slow. To that end, lawmakers should keep in mind that better choices are made when there is trust, that critiques are sophisticated and fair, and when the expectations on decisions and judgments will be guided by how they were made, not only by how they turned out. Regulatory challenges for maintaining high levels of innovation that also protect effective competition should be addressed with care. However, as the COVID-19 pandemic has shown us, maybe the time has come that those making the calls increase the use of life well-being indicators as guidance rather than traditional innovation metrics alone.

## Footnotes

1 *See*https://www.bbc.com/future/article/20200415-covid-19-could-bluetooth-contact-tracing-end-lockdown-early.

2 *See*http://infojustice.org/archives/42220.

3 *See*https://opencovidpledge.org/.

4 Taleb N (2007) *The Black Swan: The Impact of the Highly Improbable*. Random House, New York.

5 *See*https://ipaccessmeds.southcentre.int/wp-content/uploads/2020/04/Covid-19-CL-Table-FINAL.pdf.

6 Among others, *see* Mueller-Langer F (2013) “Neglected infectious diseases: Are push and pull incentive mechanisms suitable for promoting drug development research?” Health Economics, Policy and Law 8(2):185–208.

7 Gurría A (2009) “Remarks at the TransAtlantic Business Dialogue executive board”, https://www.oecd.org/fr/presse/dealingwiththecrisisreformsthatwillputusbackonapathofgrowthinnovationandsustainability.htm.

8 See Ullrich H (2012) “Intellectual Property: Exclusive Rights for a purpose – the case of technology protection by patents and copyright”, Max Planck Institute for Intellectual Property & Competition Law Research Paper No. 13-01, https://papers.ssrn.com/sol3/papers.cfm?abstract_id=2179511.

9 Ullrich H (2008) “Expansionist Intellectual Property Protection and Reductionist Compteition Rules: A TRIPS Perspective” in Ullrich H., Govaere, I. (eds.) *Intellectual Property, Market Power, and the Public Interest*, Lang, Brussels, p. 402.

10 Schwab K (2017) *The Fourth Industrial Revolution*, Crown Publishing Group, New York.

11 Desantes Real M (2020) “The Disruptive Nature of the So-Called Fourth Industrial Revolution for Intellectual Property: Moving Societal Changes from Linear to Exponential” in Heath C., Kamperman Sanders A., Moerland A. (eds.)* Intellectual Property Law and the Fourth Industrial Revolution*, Wolters Kluwer, Alphen aan den Rijn, pp. 3–28.

12 Allott P (1992) “Reconstituting Humanity – New International Law”, 3 EJIL:219–252, p. 224.

